# Gabapentin reduces CX3CL1 signaling and blocks spinal microglial activation in monoarthritic rats

**DOI:** 10.1186/1756-6606-5-18

**Published:** 2012-05-30

**Authors:** Jia-Le Yang, Bo Xu, Shuang-Shuang Li, Wei-Shi Zhang, Hua Xu, Xiao-Ming Deng, Yu-Qiu Zhang

**Affiliations:** 1 Institute of Neurobiology, Institutes of Brain Science and State Key Laboratory of Medical Neurobiology, Fudan University, Shanghai 200032, China; 2 Department of Anesthesiology, Xinhua Hospital, Shanghai Jiaotong University School of Medicine, Shanghai 200092, China; 3 Department of Anesthesiology, Changhai Hospital, The Second Military Medical University, Shanghai 200433, China

**Keywords:** α2/δ-1 subunit of Voltage-gated calcium channels, CX3CL1, CX3CR1, Gabapentin, Glial activation, Monoarthritis

## Abstract

**Background:**

Spinal glia, particularly microglia and astrocytes, are of the utmost importance in the development and maintenance of chronic pain. A recent study from our laboratory revealed that gabapentin, a recommended first-line treatment for multiple neuropathic conditions, could also efficiently antagonize thermal hyperalgesia evoked by complete Freund's adjuvant (CFA)-induced monoarthritis (MA). In the present study, we investigated whether the spinal glia are involved in the anti-hyperalgesic effect of gabapentin and how this event occurs.

**Results:**

Unilateral intra-articular injection of CFA produced a robust activation of microglia and astrocytes. These cells exhibited large cell bodies, thick processes and increases in the ionized calcium binding adapter molecule 1 (Iba-1, a microglial marker) or the glia fibrillary acidic protein (GFAP, an astrocytic marker). These cells also displayed immunoreactive signals, and an upregulation of the voltage-gated calcium channels (VGCCs) α2/δ-1 subunit, CX3CL1 and CX3CR1 expression levels in the spinal cord. These changes were associated with the development of thermal hyperalgesia. Immunofluorescence staining showed that VGCC α2/δ-1 subunit, a proposed gabapentin target of action, was widely distributed in primary afferent fibers terminals and dorsal horn neurons. CX3CL1, a potential trigger to activate microglia, colocalized with VGCC α2/δ-1 subunits in the spinal dorsal horn. However, its receptor CX3CR1 was mainly expressed in the spinal microglia. Multiple intraperitoneal (i.p.) gabapentin injections (100 mg/kg, once daily for 4 days with the first injection 60 min before intra-articular CFA) suppressed the activation of spinal microglia, downregulated spinal VGCC α2/δ-1 subunits decreased CX3CL1 levels and blocked the development of thermal hyperalgesia in MA rats.

**Conclusions:**

Here we provide the first evidence that gabapentin diminishes CX3CL1 signaling and spinal microglia activation induced by joint inflammation. We also show that the VGCC α2/δ-1 subunits might be involved in these events.

## Background

Rheumatoid arthritis (RA) is a systemic inflammatory disorder characterized by pain as the predominant clinical feature. Like other conditions that produce chronic inflammatory pain, arthritis is characterized by a heightened pain response to noxious (hyperalgesia) or innocuous (allodynia) stimulation and pain at rest (spontaneous pain). The inflammation of the joint causes an increased efficacy of synaptic transmission between the primary afferent fibers and the dorsal horn neurons (known as peripheral sensitization). This corresponds with the development of central sensitization, whereby neurons within the spinal cord also become hyperexcitable with an increased response to peripheral stimulation. It is well known that spinal glia, particularly microglia (CNS macrophages) and astrocytes, are of the utmost importance in the development and maintenance of chronic pain [1-3]. Following peripheral or central inflammation and damage, the spinal glia become activated. This activation produces changes in morphology and increases in the release of algesic substances (especially pro-inflammatory cytokines), which enhance pain transmission [1,4,5]. Activation of the spinal glia appears to correlate with the development and maintenance of behavioral hypersensitivity induced by spinal injury, peripheral nerve injury, formalin, zymosan, and complete Freund’s adjuvant (CFA) [5-10]. A series of studies from our laboratory further demonstrated that disruption of glial function with fluorocitrate (a glial metabolic inhibitor) or minocycline (a microglial inhibitor) markedly blocked thermal hyperalgesia and mechanical allodynia in CFA-induced monoarthritic rats. This disruption was associated with the ability to suppress spinal glial activation [8-11], suggesting that activated glia play a major role in mediating the behavioral hypersensitivity evoked by joint inflammation.

Gabapentin, which has only minor side effects at clinically effective doses, has been widely used in the treatment of chronic pain states [12]. In animal studies, gabapentin has also been shown to possess analgesic properties in a wide range of chronic pain models including peripheral and central neuropathic, postsurgical, and arthritic pain [12-16]. Although the mechanisms of action of gabapentin have yet to be ascertained, evidence implies that gabapentin may act at the α2/δ-1 subunit of the calcium channel [17]. Gabapentin is thought to decrease neuronal activity by binding to the α2/δ-1 subunit. This binding likely inhibits calcium currents and prevents extracellular calcium entry, which is essential for subsequent vesicular exocytosis [14,18,19]. Also, evidence shows that gabapentin is an inhibitor of calcium channel subunit trafficking [20,21]. Additionally, gabapentin is able to block the persistent sodium current (*I*_NaP_) [22] and open K^+^ channels [23], inhibiting the abnormal spontaneous activity and hyperexcitability of sensory neurons, leading to a reduction of pain in rats. A recent report showed that oral gabapentin can also significantly reverse diabetes-induced allodynia and suppress microglial activation in the spinal dorsal horn. This evidence suggests that gabapentin may exert its anti-allodynic actions partially through alterations of microglia function [24]. In the present study, we further address whether the anti-hyperalgesic effect of gabapentin in CFA monoarthritic rats is also linked to a reduction of in the activation of spinal glia. If so, the possible mechanisms by which gabapentin modulates the activation of glia would be further defined.

## Results

### Time course of MA-induced spinal glial activation and thermal hyperalgesia

**Figure 1:**
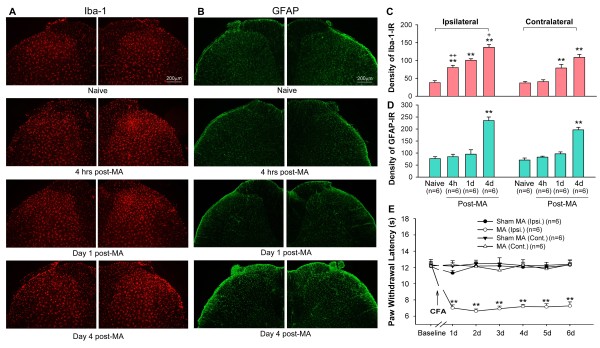
**The activation of spinal glia (microglia and astrocytes) and thermal hyperalgesia by unilateral intra-articular injection of complete Freund's adjuvant (CFA)-induced monoarthritis (MA). A**, Immunohistochemistry reveals an extensive increase in Iba-1- (a microglial marker) immunoreactivity (Iba-1-IR) in the ipsilateral lumbar spinal dorsal horn at 4 hrs and in both sides by days 1 and 4 after MA. **B**, Immunohistochemistry reveals a marked increase in GFAP- (a microglial marker) immunoreactivity (GFAP-IR) in both sides of the lumbar spinal dorsal horn on day 4 after MA. **C** and **D**, Quantification of Iba-1-IR (**C**) and GFAP-IR (**D**) expression on both sides of the spinal dorsal horn ** p < 0.01 vs. naïve control; + p < 0.05, ++ p < 0.01 vs. contralateral. E, MA induced significant thermal hyperalgesia in the ipsilateral hindpaw ** p < 0.01 vs. sham MA.

**Figure 2:**
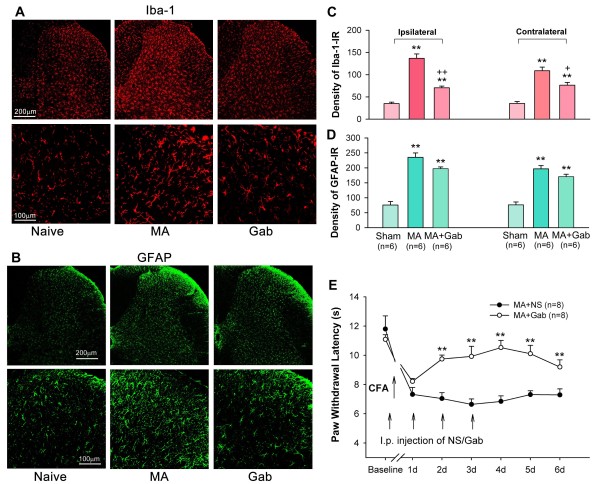
**Effects of multiple intraperitoneal (i.p.) injection of gabapentin (Gab, 100 mg/kg) on MA-induced spinal glial activation and thermal hyperalgesia.** A and B, Effects of repeated Gab treatment on MA-induced increase in Iba-1-1R (**A**) and GFAP (**B**) in the ipsilateral spinal dorsal horn. **C** and **D**, Quantification of Iba-1-IR (**C**) and GFAP-IR (**D**) expression on both sides of the spinal dorsal horn ** p < 0.01 vs. sham control; + p < 0.05, ++ p < 0.01 vs. MA. **E**, Repeated Gab treatment inhibited MA-induced thermal hyperalgesia. Repeated i.p. Gab or NS were given directly after the behavioral test on days 0–3, with the first injection 60 min before intra-articular injection of CFA. ** p < 0.01 vs. NS control.

In agreement with our previous study [8-10], the present results show an early, robust microglial activation and a delayed activation of astrocytes in the spinal cord during the CFA-induced MA. After CFA-induced MA, robust microglial activation occurred in the ipsilateral lumbar spinal dorsal horn at 4 hrs. However, on days 1 and 4 after MA, activated microglia were observed bilaterally throughout the lumbar sections of the spinal cord. Activated microglia exhibited large cell bodies and short or thick processes. The ionized calcium binding adapter molecule 1 (Iba-1, a microglial marker) and the immunoreactive (Iba-1-IR) signals were increased in MA rats (Figures 1A and 2A). A one-way ANOVA analysis showed that the density of Iba-1-IR on both sides of the spinal cord was significantly greater in MA rats than that in the controls (ipsilateral: F_3,20_ = 48.598, p < 0.01; contralateral: F_3,20_ = 19.557, p < 0.01) (Figure 1C). Unlike microglia, significant activation of the astrocytes in the spinal cord was observed 4 days after MA. Activated astrocytes exhibited intense glia fibrillary acidic protein (GFAP) immunoreactivity (GFAP-IR) and appeared to be hypertrophied with thick processes (Figures 1B and 2B). A one-way ANOVA analysis showed that the density of GFAP-IR on both sides of the spinal cord was significantly greater in the MA rats compared to the controls (ipsilateral: F_3,20_ = 29.138, p < 0.01; contralateral: F_3,20_ = 43.742, p < 0.01) (Figure 1D). 

The baseline measures of the paw withdrawal latency (PWL) to radiant heat stimulation did not differ in either hindpaw across the groups. As in our previous study [11], unilateral intra-articular injection of CFA produced marked joint inflammation (edema and erythema) and thermal hyperalgesia in the ipsilateral paw. This inflammation peaked at 1 day after CFA injection and showed little change over 6 days. Neither the PWLs of both hindpaws in the sham MA (intra-articular injection of NS) rats, nor the hindpaw contralateral to the CFA-injected joint had a significant difference before and after the intra-articular injection (Figure 1E).

### Effects of multiple i.p. gabapentin on spinal glial activation and the development of thermal hyperalgesia in CFA-induced monoarthritic rats

When repeated i.p. injections of gabapentin (100 mg/kg) were given once daily for 4 successive days (with the initial injection 1 hour before intra-articular CFA), MA-induced spinal microglial activation was significantly suppressed by day 4 (One-way ANOVA, ipsilateral: F_2,15_ = 66.27, q = 10.458, p < 0.01; contralateral: F_2,15_ = 31.67, q = 4.950, p < 0.01) (Figure 2A and C).

Repeated i.p. injections of gabapentin (100 mg/kg) caused a partial decrease in GFAP-IR on both sides of the spinal dorsal horn, although this did not reach statistical significance (One-way ANOVA, ipsilateral: F_2,15_ = 48.29, q = 3.003, p = 0.051; contralateral: F_2,15_ = 42.076, q = 2.701 p = 0.076) (Figure 2B and 2D).

Our previous study showed that a single i.p. injection of gabapentin (25, 50, 100, and 200 mg/kg) dose-dependently reversed the established thermal hyperalgesia in MA rats [15]. A study from Dickenson’s laboratory showed that chronic gabapentin treatment efficiently inhibited osteoarthritis-induced mechanical and cooling allodynia, as well as ambulatory-evoked pain [16]. To address the cumulative effects of gabapentin in the development of thermal hyperalgesia during arthritic pain, gabapentin (100 mg/kg) was repeatedly given once daily for 4 days (with the first injection 60 min before intra-articular CFA). The first injection of gabapentin before MA failed to prevent the development of thermal hyperalgesia that reliably occurred on day 1 post-MA. Following subsequent daily gabapentin administration, MA-induced ipsilateral hyperalgesia was significantly attenuated from day 2 to day 6. Three days after cessation of the i.p. gabapentin, the PWLs were still reliably greater in gabapentin-treated animals than that in NS-treated animals (Figure 2E). A two-way ANOVA analysis revealed a significant effect of the gabapentin treatment (F_1,14_ = 67.458, p < 0.01) and an interaction between the gabapentin treatment and time (F_8,84_ = 6.847, p < 0.01).

### Expression of the VGCC α2/δ-1 subunit in the spinal dorsal horn

**Figure 3:**
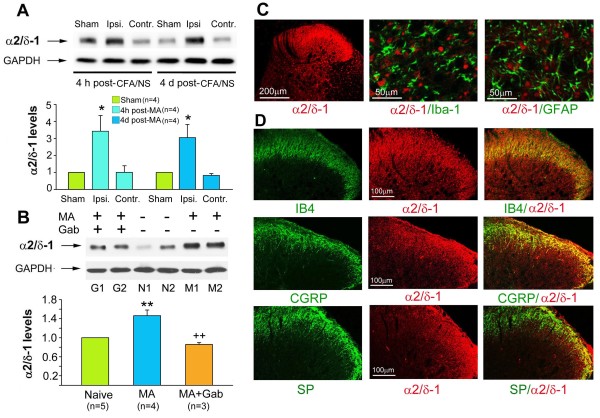
**Expression of the voltage-gated calcium channels (VGCCs) α2/δ-1 subunit in the lumbar spinal cord. A**, The western blot shows an increase in the level of VGCC α2/δ-1 subunit expression in the ipsilateral lumbar spinal dorsal horn at 4 hrs and 4 days after MA (CFA/NS injected into the ankle articular cavity). **B**, Western blot analysis reveals that repeated Gab treatment significantly suppresses MA-induced upregulation of the VGCC α2/δ-1 subunit in the ipsilateral lumbar spinal dorsal horn. * p < 0.05, ** p < 0.01 vs. control (sham MA or naïve); ++ p < 0.01 vs. MA. **C**, Immunohistochemistry reveals that the VGCC α2/δ-1 subunits are extensively distributed in the spinal dorsal horn. Double immunofluorescence staining shows that the VGCC α2/δ-1 subunit does not colocalized with the microglial marker Iba-1 or the astrocytic marker GFAP in the spinal dorsal horn. **D**, Double immunofluorescence staining shows that VGCC α2/δ-1 subunits are colocalized with IB4 in the inner lamina II and CGRP and SP in the lamina I and outer lamina II of the spinal cord.

**Figure 4:**
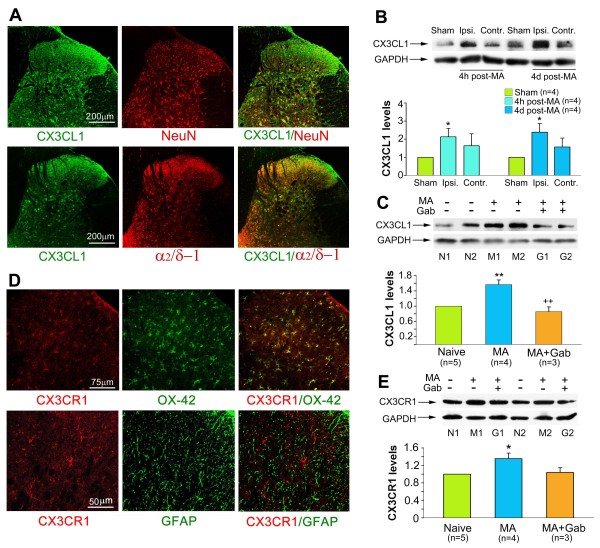
**Expression of CX3CL1 and its receptor CX3CR1 in the lumbar spinal cord. A**, Double immunofluorescence staining shows that CX3CL1 is colocalized with the neuronal marker NeuN and VGCC α2/δ-1 subunits in all of the layers of the spinal dorsal horn. **B**, The western blot shows an increase in the level of CX3CL1 in the ipsilateral lumbar spinal dorsal horn at 4 hrs and 4 days after MA. **C**, Western blot analysis reveals that repeated Gab treatment significantly suppresses MA-induced upregulation of CX3CL1 in the lumbar spinal dorsal horn. **D**, Double immunofluorescence staining shows that CX3CR1 is colocalized with the microglial marker OX-42, but not the astrocytic marker GFAP. **E**, Western blot analysis reveals that repeated Gab treatment partially inhibits MA-induced upregulation of CX3CR1 in the lumbar spinal dorsal horn. * p < 0.05, ** p < 0.01 vs. control (sham MA or naïve); ++ P < 0.01 vs. MA.

An increase in the VGCC α2/δ-1 subunit (an analgesic gabapentin target of action), in the dorsal spinal cord has been reported in diabetic- and paclitaxel-induced pain [17,25]. Consistent with this observation, the present study showed a significant increase in the expression of the VGCC α2/δ-1 subunit in the ipsilateral spinal dorsal horn at 4 hrs and 4 days after CFA-induced MA (Figure 3A). After repeated i.p. injections of gabapentin (100 mg/kg, given once daily for 4 successive days with the initial injection 1 hour before intra-articular CFA), MA-induced upregulation of the VGCC α2/δ-1 subunit was significantly suppressed by day 4 post-MA (One-way ANOVA, F_2,9_ = 18.44, q = 7.87, p < 0.01) (Figure 3B). Moreover, we examined whether the VGCC α2/δ-1 subunit is, co-expressed with Iba-1 and GFAP in the spinal dorsal horn. This experiment was to determine the possible mechanisms of gabapentin-induced inhibition in the activation of spinal glia. Unexpectedly, we found no evidence that the VGCC α2/δ-1 subunit colocalized with the microglial marker Iba-1 or the astrocytic marker GFAP in the spinal dorsal horn (Figure 3C). In contrast, the α2/δ-1-IR signals were mainly detected in the primary afferent termination of the superficial spinal cord. Thus the VGCC α2/δ-1 subunit was co-expressed with isolectin B4 (IB4, a marker of non-peptidergic C-type neurons), calcitonin gene-related peptide (CGRP) and substance P (SP, a marker of peptidergic neurons) (Figure 3D). Additionally, α2/δ-1-IR cells were observed in neurons of the deep spinal dorsal horn (Figure 3C, 4A). 

### Expression of CX3CL1 and CX3CR1 in the spinal dorsal horn

Previous studies suggested that fractalkine (CX3CL1) and its unique CX3CL1 receptor (CX3CR1) play a role in signaling between the neurons and microglia [26-28]. To further address the possible mechanisms by which gabapentin inhibits microglial activation in the spinal cord, we examined the expression of CX3CL1 and CX3CR1 in the spinal dorsal horn. CX3CL1-IR was extensively distributed in all layers of the spinal dorsal horn, including the propriospinal neurons and the primary afferent termination. Additionally, CX3CL1-IR colocalized with the neuronal marker NeuN and the VGCC α2/δ-1 subunit (Figure 4A). Following the CFA-induced MA, CX3CL1 expression levels were increased in the ipsilateral spinal dorsal horn, which was suppressed by repeated i.p. injections of gabapentin (100 mg/kg) (One-way ANOVA, F_2, 9_ =17.79, q = 7.60, p < 0.01) (Figure 4B and 4C).

CX3CR1-IR was detected in whole layers of the spinal cord. Similar to previous studies in neuropathic pain models [29,30], CX3CR1 was co-expressed with spinal OX-42-IR (another microglial marker) rather than GFAP in CFA-induced MA rats (Figure 4D). Consistent with our previous study [8], CFA induced a significant upregulation of CX3CR1 expression in the bilateral spinal dorsal horn on day 4 after MA. Repeated i.p. injections of gabapentin (100 mg/kg) caused a decrease in CX3CR1 expression in the spinal cord by day 4 post-MA, although this did not reach statistical significance (One-way ANOVA, F_2, 9_ =4.439, q = 3.111, p = 0.055) (Figure 4E).

## Discussion

The main findings of the present study are that the VGCCs α2/δ-1 subunit (a proposed gabapentin target of action), CX3CL1 and its receptor CX3CR1 (a potential trigger to activate microglia) are upregulated in the spinal cord following intra-articular injection of CFA. These upregulated proteins were associated with the activation of spinal glia and the development of thermal hyperalgesia. Gabapentin treatment attenuated MA-induced thermal hyperalgesia and activation of spinal glia. Gabapentin treatment also decreased expression of the α2/δ-1 subunit and CX3CL1. Consistent with thses observations, a previous study from Wodarski et al [24] showed that in STZ-induced diabetic neuropathy mechanical allodynia was associated with increased spinal microglial activation. In this study, gabapentin treatment significantly attenuated established mechanical allodynia and spinal microglial activation.

**Figure 5:**
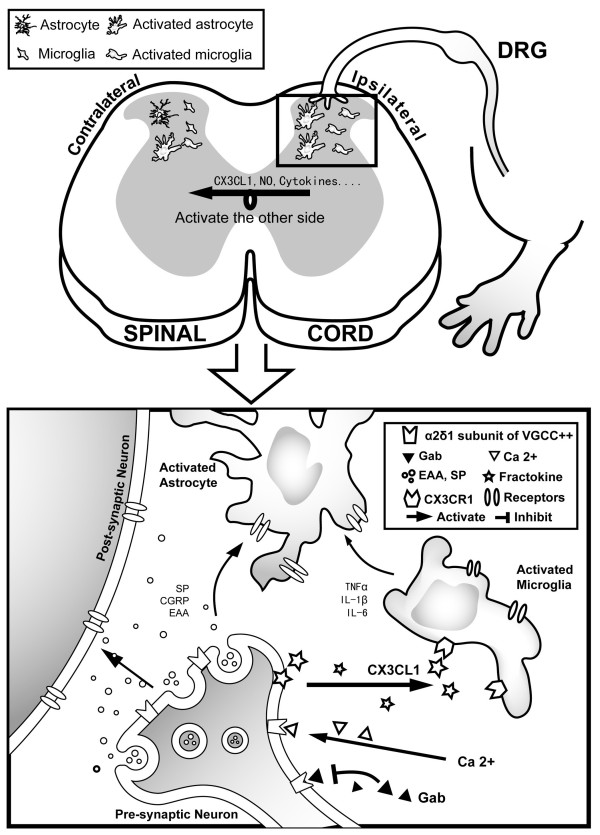
**A schematic illustration of spinal glial activation in monoarthritic pain.** Joint inflammation may increase the release of nociceptive transmitters and modulators (such as EAAs, SP, ATP and CX3CL1) in the spinal dorsal horn from the primary afferent terminals ipsilateral to the inflammed joint [39]. These events can initiate early focal microglial activation in ipsilateral spinal cord (within 4 hrs after MA). Activated microglia release several proinflammatory cytokines and chemokines that may spread to contralateral spinal cord, leading to the contralateral spinal microglial and astrocytic activation [3]. Once the glia are activated, they release several glial products including proinflammatory cytokines, tumor necrosis factor-α, and inflammatory mediators. This leads to an exaggerated release of neurotransmitters from presynaptic neurons, sensitization of the post-synaptic membrane, activation of neighboring astrocytes, and enhancement of the microglial propagation of neuromediators [5]. Such positive feedback loops sustain the perseverant release of pain mediators, facilitating the development of neuronal hypersensitivity, leading to exaggerated pain (such as thermal hyperalgesia) [13]. Gabapentin might diminish the release of “pain” neurotransmitters/neuromodulators (such as CX3CL1) and activation of microglia in the spinal cord by modulating VGCC α2/δ-1 subunits, leading to a reduction in thermal hyperalgesia.

An early microglial and delayed astrocytic activation in the spinal dorsal horn have been observed in neuropathic and inflammatory pain models [3,5,8-10]. Despite some conflicting reports [27,30], most evidence showed that spinal microglia and astrocytes were activated by intraplantar or intra-articular injection of CFA, subcutaneous injection of phospholipase A2, snake venom and formalin [8-10,31,32]. The present results showed that spinal microglia were activated ipsilaterally at 4 hrs and bilaterally at 1 day after MA. Meanwhile, activation of the spinal astrocytes was detected bilaterally at 4 day after MA. Similar to the present study, glial activation is observed bilaterally in the lumbar sections of the spinal cord in other peripheral inflammation models [3,27]. This is in contrast to peripheral neuropathy, which resulted in glial activation that was predominantly localized ipsilateral to the injury [33]. We are unable to give a clear explanation of this phenomenon at the present. The temporal and spatial sequence of microglial and astrocytic activation in the spinal cord after MA suggest that glialexcitatory substances from the focal ipsilateral microglia response in the early phase (such as 4 hrs after MA in the present study) may spread to the contralateral spinal cord (Figure 5). The proinflammatory cytokines TNF-α, IL-1β and IL-6 are generally considered to play key roles in mediation of this event [6,34]. Our previous study demonstrated a bilateral upregulation of TNF-α, IL-1β and IL-6 mRNA by MA [9]. Additionally, astrocytes are organized in gap junction-coupled networks, which could transmit Ca^2+^ signaling in the form of oscillations or waves through the networks [35,36]. Thus, it is possible that astrocyte networks could mediate the spread. Another explanation is that the bilateral glial response to inflammation results from systemic or perivascular signals [37]. It has been demonstrated that in inflammatory pain, there is a partial opening in the tight junctions between the endothelial cells of the capillaries, increasing the blood–brain barrier permeability [38]. 

It is well known that activated microglia and astrocytes release several proinflammatory cytokines, chemokines, and other neuro- and glial-excitatory substances. These substances facilitate pain processing by further enhancing glial activation, presynaptic release of neurotransmitters and postsynaptic excitability. Such positive feedback loops sustain the persistent release of pain mediators, facilitating the development of neuronal hypersensitivity, which leads to hyperalgesia and allodynia [1,5,7,13]. Interestingly, although spinal glial activation was observed in the dorsal horn contralateral to the MA, the contralateral hind paw nociceptive thresholds remained unchanged. Indeed, bilateral increases in neuropeptide synthesis [40] and phosphorylation of the cAMP-responsive element-binding protein (CREB) [41] have been reported in the spinal cord following unilateral injection of CFA and formalin, respectively. These events were shown to produce hyperalgesia ipsilaterally. Additionally, several reports have shown that spinal glial activation and enhanced pain states are disassociated when determined by OX-42 or Iba-1 (microglia) and GFAP (astrocyte) immunoreactivity. For example, Colburn et al. observed that in some rats pain behaviors existed in the apparent absence of microglial activation. Conversely, a profound microglial response was occasionally associated with a lack of pain behaviors [33]. Zhuang et al. showed that a JNK inhibitor reversed neuropathic pain for several days without an accompanying reduction of GFAP expression [42]. Our present study showed that MA resulted in a bilateral upregulation of Iba-1 and GFAP expression in the spinal cord without contralateral thermal hyperalgesia. Additionally, gabapentin attenuated thermal hyperalgesia and Iba-1 expression but did not significantly suppress GFAP expression. Iba-1 or OX-42 and GFAP are the most widely used markers for demonstrating microglial and astrocytic activation at present. However, whether Iba-1 and GFAP contribute to pain hypersensitivity per se remains unclear. Thus, more sensitive markers of nociceptive changes and glial activation need to be examined in further studies. An alternative explanation for the contralateral side, which did not present obvious hyperalgesia may be that the contralateral hind limb becomes a load-bearing limb when the unilateral knee joint suffers from arthritis. Thus, unilateral load-bearing may affect the measurement of PWLs on free-moving rats.

The glia are sensitive to many types of disturbances in the nervous system. Tissue/nerve inflammation/damage leads to the release of nociceptive transmitters and modulators, including excitatory amino acids (EAAs), CGRP, substance P (SP), ATP and NO from the primary afferent neurons [39]. All of the “pain” neurotransmitters/modulators can cause glial activation via the corresponding receptors expressed on the spinal microglia and astrocytes (such as NMDA, AMPA/KA, NK-1, P2X4 and P2X7 etc.) [43]. It has been shown that gabapentin is capable of reducing the release of peripheral inflammation or nerve injury-induced spinal EAAs (glutamate and aspartate) [44] and neuropeptides (SP and CGRP) [45]. Gabapentin also inhibits the enhancement of K^+^- or SP- and CGRP-evoked glutamate release in caudal trigeminal slices [46]. Consistent with the inhibition of spinal glutamate release, gabapentin markedly reduces miniature EPSCs in spinal cord slice [47]. Thus, one of the mechanisms by which gabapentin suppresses MA-induced spinal glial activation, might be by diminishing the release of the “pain” neurotransmitters/neuromodulators from the primary afferent terminals and spinal nociceptive neurons. In the present study, the reduction of spinal microglial marker by gabapentin is unlikely due to a direct action on glia because no colocalization of the VGCCs α2/δ-1 subunit (a binding target of gabapentin) and Iba-1 (a microglial marker) was found in the spinal dorsal horn. In contrast, double staining signals of the VGCCs α2/δ-1 subunit and IB4 (a marker of non-peptidergic C-type neurons) or CGRP and SP (markers of peptidergic neurons in superficial spinal dorsal horn) were observed. It was also reported that mRNA levels of the VGCCs α2/δ-1 subunit were increased in the DRG in osteoarthritis [48]. This finding is consistent with the distribution of the α2/δ-1 subunit in the primary afferent terminals of the spinal cord. It should be mentioned that gabapentin was also reported to block the persistent Na^+^ current in DRG neurons at relatively low concentrations (compared to doses achieved clinically). Importantly, this blockage occurred at a somewhat lower dose than required to inhibit the Ca^2+^ channel in DRG neurons, suggesting that there is another analgesic action target of gabapentin [22]. This could occur if gabapentin suppressed the abnormal spontaneous activity and DRG hyperexcitability, resulting in an attenuation of spinal glial activation.

Fractalkine (CX3CL1) is a unique chemokine in the central nervous system (CNS), which acts exclusively on the CX3CL1 receptor (CX3CR1) [49]. Intriguingly, both in the spinal cord and dorsal root ganglion (DRG), CX3CL1 immunoreactivity and mRNA were observed in neurons. However, CX3CR1 immunoreactivity and mRNA were found to be mainly restricted to the OX-42- and CD4-positive microglia in the spinal dorsal horn [27,29,30]. This led to the idea of CX3CL1 as a critical mediator of spinal neuronal-microglial communication in chronic pain [26,28]. Following peripheral nerve injury and inflammation, CX3CR1 knockout mice showed deficits in inflammatory and neuropathic nociceptive responses. In contrast, wild-type mice showed significant thermal hyperalgesia and mechanical allodynia. This was associated with upregulated CX3CL1 and CX3CR1 levels and increased Iba-1 immunostaining as well as phosporylation of p38 MAPK in the spinal cord [50]. Consistent with this data, the present study showed that CX3CL1 immunoreactivity was detected in all layers of the spinal dorsal horn. CX3CL1 also colocalized with the VGCC α2/δ-1 subunit and almost all of the CX3CR1-positive cells were OX-42 (a microglial marker)-positive cells. Moreover, following CFA-induced MA, expression of the VGCC α2/δ-1 subunit, CX3CL1 and CX3CR1 in the spinal dorsal horn were increased. This increase in expression was attenuated by gabapentin. A discrepancy is that MA induced unilateral upregulation of the VGCC α2/δ-1 subunit, upregulation of CX3CL1 and bilateral activation of microglia and astrocytes. We presume that peripheral inflammation may increase the release of nociceptive transmitters and modulators (such as EAAs, SP, ATP and CX3CL1) in the spinal dorsal horn. This may occur via the upregulated VGCC α2/δ-1 subunits on the primary afferent terminals ipsilateral to inflammatory arthritis. This initiates early focal microglial activation in the ipsilateral spinal cord (within 4 hrs after MA). Activated microglia release several proinflammatory cytokines and chemokines that may spread to the contralateral spinal cord, leading to contralateral spinal microglial and astrocytic activation. Actually, a trend towards increased CX3CL1 expression was observed in the spinal cord contralateral to the MA (Figure 4B). Additionally, our previous study revealed a bilateral upregulation of CX3CR1 in the spinal dorsal horn [8]. Preemptive and early consecutive treatments of gabapentin may block microglial activation by modulating VGCC α2/δ-1 subunits to attenuate the release of nociceptive transmitters and modulators (including CX3CL1) in the spinal cord (Figure 5).

## Conclusions

Gabapentin treatment attenuated MA-induced thermal hyperalgesia and spinal glial activation. The reduction of spinal glial activation by gabapentin is likely to be due to an indirect action on the glia by modulation of the VGCC α2/δ-1 subunits. This modulation may diminish the release of the “pain” neurotransmitters/neuromodulators (including CX3CL1) from primary afferent terminals and spinal nociceptive neurons.

## Methods

### Animals

The experiments were performed on adult male Sprague Dawley rats (Experimental Animal Center, Shanghai Medical College of Fudan University, China) weighing 180–220 g. The animals were housed in a temperature-controlled (22 ± 2°C) and light-controlled (12-h dark /12-h light cycle) room with free access to food and water. All of the experimental protocols and animal handling procedures were approved by the Animal Care and Use Committee of Fudan University and were consistent with the National Institutes of Health Guide for the Care and Use of Laboratory Animals. All efforts were made to minimize the number of animals used and their suffering.

### Drugs

Gabapentin (Jiangsu Hengrui Medicine CO. LTD) was diluted in normal saline (NS, 0.9% NaCl). It was administered i.p. in a dose of 100 mg/kg body weight [14]. Control animals received an equivalent volume of sterile NS.

### Induction of monoarthritis

Monoarthritis (MA) was induced by injection of complete Freund's adjuvant (CFA) into the unilateral ankle articular cavity [11]. The rat was briefly anesthetized with isoflurane. The skin around the site of injection was sterilized with iodine tincture and 75% alcohol. The right leg of the rat was held and the fossa of the lateral malleolus of the fibula was located. A 28-gauge needle was inserted vertically to penetrate the skin and turned distally to insert into the articular cavity from the gap between the tibiofibular and tarsus bone, until a distinct loss of resistance was felt. A volume of 50 μl of CFA was then injected. Sham MA control animals were similarly injected with sterile NS.

### Hargreaves’ test for thermal hyperalgesia

After acclimation to the test chamber, thermal hyperalgesia was assessed by measuring the latency of paw withdrawal in response to a radiant heat source. The rats were housed individually in Plexiglas chambers on an elevated glass platform under a radiant heat source (Model 336 Combination Unit, IITC/life Science Instruments, Woodland Hill, CA, USA). The heat was applied to the plantar surface of the hind paw through the glass plate. The heat source was turned off when the rat lifted the foot, allowing measurement of the time from the onset of radiant heat application to withdrawal of the rat’s hindpaw. This time was defined as the paw withdrawal latency (PWL). The heat was maintained at a constant intensity, which produced a stable PWL of approximately 10–12 s in the absence of arthritis. A 20 s cutoff was used to prevent tissue damage. Both hindpaws were tested independently with a 10 min interval between trials [8].

### Immunohistochemistry

After defined survival times, rats were given an overdose of urethane (2 g/kg, i.p.) and perfused intracardially with saline followed by 4% paraformaldehyde in 0.1 M phosphate buffer (PB, pH 7.4). The L4/5 segments of the spinal cord were then removed, post-fixed in the same fixative for 4 h at 4°C, and immersed from 10% to 30% gradient sucrose in PB for 24–48 h at 4°C for cryoprotection. Transverse spinal sections (35 μm) were cut in a cryostat and processed for immunofluorescence. All of the sections were blocked with 10% donkey serum in 0.01 M phosphate buffered saline (PBS, pH 7.4) with 0.3% Triton X-100 for 2 h at RT. The samples were then incubated overnight at 4°C with rabbit anti-Iba-1 (1:2000, Serotec), mouse anti-calcium channel (α2/δ-1 subunit) (1:500, LifeSpan Biosciences) or rabbit anti-glial fibrillary acidic protein (GFAP, 1:2000, Sigma) primary antibodies in PBS with 1% normal donkey serum and 0.3% Triton X-100. Following three 10 min rinses in 0.01 M PBS, the sections were incubated with fluorescein isothiocyanate (FITC)-conjugated donkey anti-mouse IgG (1:200, Jackson Immunolab) or rhodamine-conjugated donkey anti-rabbit IgG (1:200, Jackson Immunolab) for 120 min at 4°C, then washed with PBS. For the α2/δ-1/GFAP, α2/δ-1/Iba-1, α2/δ-1/CGPR, α2/δ-1/IB4, α2/δ-1/SP, α2/δ-1/CX3CL1, CX3CR1/OX-42 and CX3CR1/GFAP double immunofluorescence, the sections were incubated with a appropriate mixtures of the following antibodies overnight at 4°C: mouse anti-α2/δ-1 and rabbit anti-GFAP, rabbit anti-Iba-1, goat anti-CGRP (1:500, Santa Cruze), rabbit anti-SP (1:100, Sigma), goat anti-CX3CL1 (1:200, R&D system), or rabbit anti-CX3CR1 (1:1000, Abcampare; goat anti-CX3CR1, 1:2000, Santa Cruz Biotechnology) and mouse anti-NeuN (1:5000, Millipore), mouse anti-OX-42 (1:3000, Serotec), or mouse anti-GFAP (1:2000, Sigma). This was followed by incubation with a mixture of FITC- and rhodamine red-X-conjugated secondary antibodies or FITC-conjugated IB4 (1:1000, Sigma) for 2 h at RT. The specificity of immunostaining was verified by omitting the primary antibodies. The specificity of the primary antibodies was verified by the preabsorption experiment (control peptide for CX3CL1 from Acris Antibodies; for CX3CR1 from Abcampare and Santa Cruz; for α2/δ-1 from LifeSpan). All of the sections were coverslipped with a mixture of 50% glycerin in 0.01 M PBS, and then observed with a Leica SP2 confocal laser-scanning microscope. Because the morphology of both microglia and astrocytes is complex and immunoreactive staining includes both cell bodies and their processes, cell counts may not sufficiently quantify activation. Therefore, the optical density of immunoreactive staining for Iba-1 or GFAP was measured with the Image J analysis system [51]. For each animal, six to eight sections of spinal L4 − 5 sections were randomly selected for quantitative evaluation. The corrected density values of the sections were averaged to provide a mean density for each animal.

### Western blot analysis

After defined survival times, rats were killed by an overdose of urethane (SCR Co., Shanghai, China). The L4-5 spinal cord from naive, MA or gabapentin-treated rats was rapidly removed. Then the spinal segment was cut into a left and right half from the ventral midline. Finally, the right half was further split into the dorsal and ventral horn at the level of the central canal. After dissection, all of the tissues were rapidly frozen in liquid nitrogen and stored at −70°C until further processing.

Frozen spinal cords were directly homogenized in a lysis buffer (12.5 ml/mg tissue) containing a cocktail of protease inhibitors and PMSF (Sigma). After centrifugation at 10,000 rpm for 15 min, the supernatant was used for western blot analysis. Equal amounts of protein (20 mg) were loaded into each lane and separated by 10% SDS–PAGE. The resolved proteins were transferred onto PVDF membranes. The membranes were blocked in 10% nonfat dry milk for 2 h at room temperature (RT). The membranes were then incubated overnight at 4°C with mouse anti-α2/δ-1 (1:100), goat anti-CX3CL1 (1:1000), rabbit anti-CX3CR1 (1:1000) or mouse anti-GAPDH (1:10000, Sigma) primary antibodies. The blots were incubated for 2 h at RT with horseradish peroxidase (HRP)-conjugated donkey anti-mouse, donkey anti-goat or donkey anti-rabbit secondary antibodies (1:5000, Santa Cruz Biotechnology). The signals were visualized using enhanced chemiluminescence (ECL, Pierce) and exposed onto X-films for 1–10 min. Pre-absorption of the primary antibodies with the blocking peptide served as specificity control. All western blot analyses were performed at least three times, and parallel results were obtained. X-ray films with blotting bands for each sample from different rats were scanned, and the density of the band area was quantified with a method described by Sun et al. [8]. The same size square was drawn around each band to measure the density, and the background near that band was subtracted. GAPDH expression was used as a loading control for protein expression. The expression level of the proteins is an average of the densities per band area from different rats that were treated.

All of the behavioral testing and quantification of immunohistochemical and western blot experiments were performed blindly, with respect to the treatments.

### Statistical analysis

The data are given as the mean ± standard error of the mean (S.E.M.). Both the pre-MA baseline and pre-drug treatment measures were analyzed by a one-way analysis of variance (ANOVA). The post-drug time course measures for hyperalgesia were analyzed by a two-way RM ANOVA (treatment × time) followed by a Newman–Keuls post hoc test. Immunohistochemical and western blot analysis were performed using Student’s t-test when comparing two groups, or a one-way ANOVA followed by Dunnett’s test for multiple comparisons when comparing more than two groups. A value of *p* < 0.05 was considered statistically significant.

### Experimental design

Experiment 1 was designed to test the effects of repeated i.p. injection of gabapentin on MA-induced thermal hyperalgesia. After the baseline behavioral assessments, the rats received an intra-articular injection of 50 μl CFA or sterile NS (day 0). Repeated i.p. injections of gabapentin (100 mg/kg) or vehicle (NS) were carried out once daily for 4 days after behavioral testing. The first injection was given 60 min before the intra-articular injection of CFA. A Hargreaves’ test was performed from days 1 to 6. Experiment 2 was designed to test MA-induced changes in spinal GFAP, Iba-1, VGCC α2/δ-1 subunit and CX3CL1 expression levels. Rats received an intra-articular injection of 50 μl CFA or sterile NS. The rats were then sacrificed at 4 hrs, 1 and 4 days after CFA injection for immunohistochemical or western blot processing. Experiment 3 was designed to test the effects of repeated i.p. injection of gabapentin on MA-induced changes in GFAP, Iba-1, VGCC α2/δ-1 subunit, CX3CL1, and CX3CR1 expression levels in the lumbar spinal cord.

## Competing interests

The authors do not have any competing interest to declare.

## Authors’ contributions

Yang JL and Xu B performed the behavioral tests, western blot and immunofluorescence experiments and drafted the manuscript. Li SS participated in the behavioral tests. Zhang WS, Xu H and Deng XM participated in producing the statistical analysis and experimental design. Zhang YQ conceived the study, designed the experiments, wrote the paper and coordinated the authors. All of the authors read and approved the final manuscript.
